# Importance-Performance Analysis of Health Perception among Korean Adolescents during the COVID-19 Pandemic

**DOI:** 10.3390/ijerph18031280

**Published:** 2021-01-31

**Authors:** Seung-Man Lee, Wi-Young So, Hyun-Su Youn

**Affiliations:** 1 Department of Physical Education, College of Education, Korea University, Seoul 02841, Korea; lsm14pe@korea.ac.kr; 2 Sports and Health Care Major, College of Humanities and Arts, Korea National University of Transportation, Chungju-si 27469, Korea; 3 Department of Physical Education, College of Education, WonKwang University, Iksan-si 54538, Korea

**Keywords:** adolescents, health perception, importance-performance analysis, COVID-19 pandemic

## Abstract

This study assessed the health perceptions of 333 Korean adolescents during the coronavirus disease (COVID-19) pandemic via an online questionnaire administered in October 2020, which queried the perceived importance and actual performance of health behaviors. The health perception scales used in the survey consists of the six dimensions of mental health, disease, physical activity, sleep, diet, and sanitary health. The data were primarily analyzed using paired sample t-test for analysis of difference and importance-performance analysis (IPA). The IPA results were presented in four quadrants—“keep up the good work,” “concentrate here,” “low priority,” and “possible overkill.” The results indicated that first, there was a positive relationship between the importance and performance of all the subdimensions of health perception. Second, sanitary healthcare was rated as being of the greatest importance and was performed most, while physical activity management was rated least important and performed least. Third, statistically significant differences were found between importance and performance for all items of mental health, disease, physical activity, sleep, and diet dimensions, and some differences were found for items assessing the hygiene control dimension. Fourth, in the two-dimensional IPA model, “sanitary health” and “disease” are in Quadrant I (keep up the good work); “mental health,” in Quadrant II (concentrate here); and “physical activity,” “sleep,” and “diet,” in Quadrant III (low priority). No components of healthcare were in Quadrant IV (possible overkill). Based on these results, we emphasize the importance of adolescent health education and discuss solutions to enhance the performance of healthcare activities.

## 1. Introduction

After the first outbreak of coronavirus disease (COVID-19) was reported in late 2019, it quickly spread globally, and on 11 March 2020, the World Health Organization (WHO) declared COVID-19 a pandemic [1]. Ever since this proclamation, COVID-19 has had worldwide political, economic, and social repercussions, along with unprecedented effects on culture and education. Experts predict that even if the pandemic ends, it will be difficult to return to life as before [2]. The biggest changes since the pandemic have been social distancing and the practice of avoiding contact. This lifestyle is expected to continue in the post-COVID-19 period [3].

People’s perception of health has also changed. Due to the pandemic, people worldwide are once again recognizing the importance and value of health; healthier lifestyle habits are being adopted based on socially distant lifestyles [2,3]. The pandemic notwithstanding, living a healthy life has been a recent major focus. As economies worldwide continue to grow, people have sought to improve their quality of life, becoming more attentive to health. The WHO states that “health is a complete state of physical, mental and social wellbeing and not merely the absence of disease or infirmity,” and the meaning of health is being expanded quantitatively and qualitatively [4].

In general, health is a condition characterized by happiness and a physical state that supports survival. Being unhealthy may result in a person being an incapable and inactive member of society, leading to a loss of positive life experiences. Moreover, when a person is “healthy,” not only do they have less anxiety regarding the pursuit of health, but they can also adapt well to various environments [5]. As such, health status depends on genetic and environmental factors, healthcare benefits, and lifestyle; consequently, it is affected by one’s social or cultural environment [6]. Therefore, health status cannot be measured without considering a range of health aspects: a person is generally considered healthy when they are not ill, have normal physical functions, are energetic (experience restful sleep and appetite), exhibit stable weight, feel comfortable (not feeling any discomfort or pain), demonstrate emotional stability, and experience harmony in their social life [6].

Meanwhile, the health sectors of developed countries are shifting their paradigm from treatment to prevention and, further, from prevention to management. Maintaining healthy habits during childhood to lay the foundation for lifelong health and disease prevention is emphasized. This necessitates promoting individuals’ interest in health by highlighting healthy habits as the foundation of lifelong wellness is necessary. Health may be perceived unequally, based upon its interpretation [7]. Providing suitable health education during the rapid growth of adolescence is likely to be more effective compared to other periods. Therefore, the question arises as to how well-informed adolescents are about health, especially during the current pandemic, and how they practice health-related matters. It is of paramount importance to educate growing adolescents on nurturing healthy lifestyles. In particular, its importance was reaffirmed in the context of the recent health crisis caused by the COVID-19 pandemic. It is believed that findings regarding the importance of adolescent health education can be used regardless of region and country. Therefore, this study on health perceptions among adolescents was considered necessary as it can provide basic data for diagnosing the status of adolescent health education and setting future directions by investigating the importance and performance of health perception for adolescents.

Importance-performance analysis (IPA) of health outcomes has been used to indicate the importance and performance of health services [8,9,10]. Rau et al. [11] performed an IPA of personal health records. IPA studies of health have produced valuable results for patients, but studies have been limited to specific subjects and situations. Given the shortcomings of these prior studies and the fact that healthy lifestyle habits in adolescence can lead to lifelong health, a global pandemic is an ideal time to investigate the health perceptions of adolescents. In this study, we divided the health perception of Korean adolescents into subdimensions such as mental health, disease, physical activity, sleep, diet, and sanitary health. Subsequently, an empirical IPA was performed, from which we assessed the implications of the relationship between importance and performance as perceived by adolescents. The findings provide an important basis for planning and strategically prioritizing future implementation of health education in public and private educational institutions.

## 2. Methods

### 2.1. Participants

In quantitative studies, a sample size of 15 times or more per observed variable is required [12]. Given that there are a total of 12 observed variables used in this study and accounting for the possibility of insincere responses, a total of 350 participants were selected. The participants comprised adolescents between the ages of 14 and 16 years who are enrolled in middle schools located in Seoul and were selected using a convenient sampling method. Among them, data of 333 participants were used in the analysis, excluding the data of 17 insincere respondents. In October 2020, a Google form (research explanation form, consent, and questionnaire) was created on the identified research topics and was sent to adolescent students through email. The demographic characteristics of the study participants are shown in Table 1. This study was conducted after obtaining ethical approval from the Institutional Review Board of Wonkwang University (WKIRB-202009-SB-053).

### 2.2. Instruments

The following demographic characteristics were investigated: sex, age, academic achievement grade, and physical activity promotion system (PAPS). We also used Ware’s “health perception” scales [7], whose reliability and validity have been verified in previous studies [13,14,15]. In particular, similar to this study, in a previous study [15] targeting Korean adolescents, reliability was verified using Cronbach’s α for the health perception scale, and confirmatory factor analysis was conducted to verify convergent validity and discriminant validity. Health perception was measured using six subdimensions: mental health (6 items), disease (3 items), physical activity (4 items), sleep (4 items), diet (3 items), and sanitary health (9 items). Responses to these items in the questionnaire were provided using a five-point Likert scale (5 = definitely agree, 4 = agree, 3 = neutral, 2 = no, 1 = not at all). Each score was calculated independently for each item.

### 2.3. Reliability and Validity of the Instruments

Cronbach’s α values of the subdimensions of health perception ranged from 0.713 to 0.921(Acceptance Criteria > 0.6, [16]), indicating high internal consistency. The study was conducted after deleting two questions (on mental healthcare importance; #4 and #5) since “alpha if item deleted” was higher than Cronbach’s α of the whole scale. The reliability for each variable used in this study is shown in Table 2.

### 2.4. Procedure and Data Analysis

We conducted an online survey (via Google form) of 333 adolescents in South Korea in October 2020. To analyze the collected data, SPSS software version 18.0 (IBM Corp., Armonk, NY, USA) was used. First, a frequency analysis was conducted to examine the demographic characteristics (sex, age, academic achievement grade, PAPS) of the participants. Second, the reliability of the research tool was verified using Cronbach’s α. Third, a correlation analysis was conducted to verify the relationships between the subdimensions of health perception. Fourth, paired-sample t-tests were conducted to assess the degree of health perception and to analyze the difference between importance and performance for each variable. Fifth, an IPA was conducted to verify the importance and performance of each variable.

IPA involves the following procedures: (1) determining what attributes to measure, (2) separating the important measures and the performance measures, (3) positioning the vertical and horizontal axes on the grid, and (4) analyzing the importance-performance grid [17]. Specifically, we measured and evaluated the degree of importance and performance of each factor in terms of health awareness; the results are indicated on the quadrant of the importance-performance matrix, and the significance of the results are expressed in terms of status maintenance, intensive improvement, inferior priority, and avoidance of overinvestment according to their location on the grid. Moreover, statistical significance was set at *p* < 0.05.

## 3. Results

### 3.1. Descriptive Analysis

The mean, standard deviation, skewness, and kurtosis were generated for the subdimensions of health perception, as shown in Table 3. Means ranged from 3.62 to 4.76 and standard deviations from 0.43 to 0.95. Skewness ranged from 0.34 to 2.60 and kurtosis from 0.01 to 7.92, both of which were within acceptable ranges (skewness: ±3.0, kurtosis: ±10.0) [18,19]. The healthcare components considered the most important by the participants were: sanitary healthcare, followed by disease, mental health, sleep, diet, and physical activity. The ranking of the performance of these components, in decreasing order, was as follows: sanitary health, disease, mental health, diet, sleep, and physical activity.

### 3.2. Correlations

Correlations between the subdimensions of perceived health are shown in Table 4 and Table 5. There was a significant positive relationship between the importance of all subdimensions of health perception. Correlation coefficients ranged between 0.472 and 0.711; thus, there were no strong correlations (>0.800).

There was a positive relationship between the performance of each dimension of health perception, all of which were statistically significant. Correlation coefficients ranged between 0.343 and 0.648; none of the correlations were strong (>0.800).

### 3.3. Analysis of Differences between Importance and Performance of Dimensions

To evaluate the differences between the importance and performance of the subdimensions of the health of South Korean adolescents, paired-sample t-tests were conducted; the results are shown in Table 6. Significant differences were found between importance and performance for all items of mental health, disease, physical activity, sleep, and diet dimensions; some items differed significantly for sanitary health. For all items, the reported scores of importance were higher than that of performance.

“Rank” in Table 3 shows the relative ranking of each factor for the difference between importance and performance. As shown under “Rank” in Table 3, the largest mean difference between importance and performance among the six dimensions of health perception was found for sleep followed by physical activity, mental health, diet, disease, and sanitary health.

### 3.4. Analysis of Importance-Performance

To conduct the importance-achievement assessment of adolescents’ health perception in a pandemic situation, the IPA matrix is schematically created using the IPA method to identify the following: maintain the status quo (Quadrant I), intensive improvement (Quadrant II), inferior priority (Quadrant III), avoid overinvestment (Quadrant IV).

**Figure 1:**
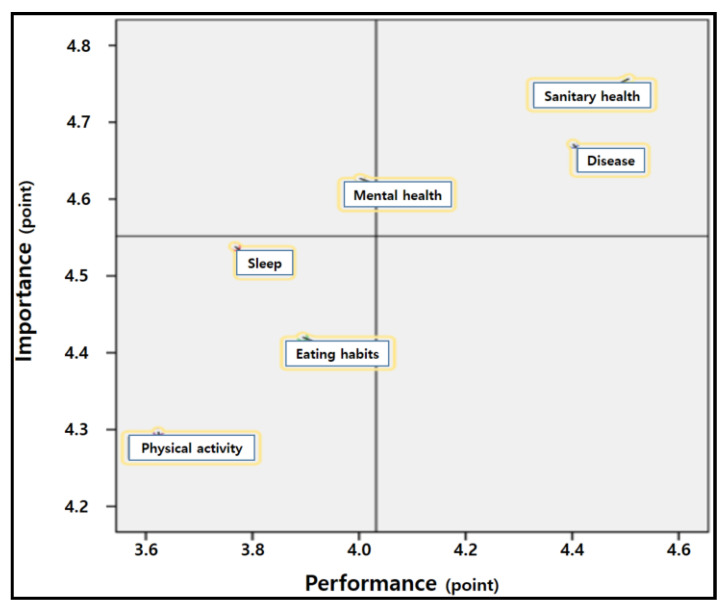
Importance-performance analysis matrix.

In Table 7 and Figure 1, the median importance value of 4.55 and the median performance value of 4.03 were used to divide the IPA plot into four quadrants.

Quadrant I (“keep up the good work” [20]) was characterized by high importance and performance; sanitary health and disease were considered important and performed frequently. Quadrant II (“concentrate here” [20]) was characterized by high importance but low performance, indicating areas that needed urgent improvement. In this quadrant, mental health was considered important, but this was not realized in actual performance. Quadrant III (“low priority” [20]) reflected low importance and performance, denoting health areas that did not require greater effort than that currently expended. Physical activity, sleep, and diet was plotted on this quadrant. Quadrant IV (“possible overkill” [20]) is characterized by low importance but high-performance, but none of the subdimensions fell on this quadrant.

## 4. Discussion

In this study, the IPA method was used to understand the importance adolescents attach to health and whether this was reflected in their healthcare performance. First, there was a statistically significant positive relationship between the importance and performance of all subdimensions of health perception. Participants perceived sanitary healthcare greatest in importance and reported the greatest performance for this aspect of health; in contrast, the lowest ratings were provided for physical activity. These rankings reflect the health perception of adolescents during the COVID-19 pandemic. It can be interpreted that preventive health behaviors such as physical activity, sleep, and eating habits, are perceived as important in normal situations, but in crisis situations such as a pandemic, direct health interventions such as disease and sanitary health are considered more important. However, when preparing for the ongoing risk of exposure to COVID-19, preventive factors must be recognized and practiced, such as engaging in physical activity, obtaining adequate sleep, and eating nutritious food, along with therapeutic approaches.

Next, differences were found between the importance and performance of all dimensions of health perception. Significant differences were found for all items of mental health, disease, physical activity, sleep, and diet dimensions, and some item-level differences were found for sanitary health. Additionally, among the subdimensions of health perceived by youth, the mean difference between importance and performance was largest for sleep, followed by physical activity, mental health, diet, disease, and sanitary health. This may reflect a difficulty in practicing healthcare activities, although Korean adolescents are aware of their importance. In particular, efforts need to be made to reduce the discrepancy between importance and performance for the subdimensions of sleep, physical activity, and diet. Meanwhile, among the subdimensions of sanitary health, “not going to public facilities” and “must wear a mask when visiting medical institutions” did not differ significantly in importance and performance; therefore, these aspects were likely considered important and are actually practiced. As such, adolescents appear to have effectively internalized the Korean government’s quarantine policies and quarantine education provided at school.

In the IPA matrix, the two dimensions of sanitary health and disease fell into Quadrant I (“keep up the good work”). This quadrant was characterized by both awareness and performance. Dimensions that fall within this quadrant can be interpreted as being successfully implemented; thus, maintaining these behaviors would represent success [21]. This may be the effect of the South Korean government’s quarantine policy and continuous education, which represents an effective approach to ensuring that the country is free of COVID-19. This policy presents direct solutions that can prevent the spread of COVID-19, such as wearing a mask, practicing social distancing, washing hands, and self-quarantining. These policies and education have made South Korean youth aware of the importance of hygiene and executing appropriate health-preserving behaviors.

The dimension of mental healthcare fell into Quadrant II (“concentrate here”). This dimension requires the most immediate improvement in performance in the future, given its perceived high importance but low performance [21]. Given an unexpected pandemic, adolescents have been unable to perform their normal activities, such as exercising, meeting friends, and engaging in hobbies; rather, they are living in limited spaces, such as homes and schools. With many adults struggling with a depression termed “corona blues,” adolescents are further likely to be subject to poor mental health. In this regard, Jefsen et al. [22] argued that children and adolescents might be particularly vulnerable to mental distress, not only because of their fear of the virus but also because of the significant social changes (social distance and isolation) initiated to minimize the spread of the virus. An editorial [23] emphasized the value of traditional therapeutic methods, such as meditation and yoga, to manage one’s own life and develop individual abilities to contribute to society. Primarily, active social intervention is needed to prevent and mitigate the negative effects of the pandemic on adolescents’ mental health [3]. To facilitate such prevention and management, empirical research on various training methods and the delivery of media that can be easily utilized by adolescents is needed. Schools, families, and communities need to generate detailed measures to address mental health issues through education and counseling.

The three dimensions of physical activity, sleep, and diet fell into Quadrant III (“low priority”). These represent dimensions that were considered low in both importance and performance to a limited degree but do not require notable further promotion [19]. During the COVID-19 pandemic, adolescents consider preventive healthcare activities like engaging in physical activity, getting proper sleep, and eating healthily to be a relatively low priority, even though such activities are important to treat disease and maintain and promote health [15,24,25]. There exist reports that younger people tend to neglect health [26]. Therefore, the Korean government has been supporting the daily healthcare of adolescents by introducing free school meals, school sports clubs, and “Weclass” in schools. However, these efforts have been hindered by the COVID-19 pandemic. There are negative factors arising from the unstable provision of school meals due to school hours being reduced by a third, the shift to online classes, wearing masks, lack of physical activity due to social distancing, smartphone addiction, and poor sleep quality caused by excessive online classes. These effects were likely reflected in the health perceptions of the adolescents.

No factor fell into Quadrant IV (“possible overkill”). This area would represent the need to transfer efforts to other dimensions due to over-emphasis on unimportant components [18]. This result represents a positive phenomenon whereby adolescents are correctly aware of the importance of health and are not engaging in unnecessary efforts. We can infer that this is due to the fact that health education in schools has been effectively conducted, and the importance of health has further been strengthened in the face of a pandemic.

Finally, we recognize the limitations of the present study and present suggestions for future research, focusing on difficulties in conducting the research and areas where control was difficult. First, it will be difficult to generalize the results of the study because our results are limited to adolescents in South Korea. Subsequent studies will need to expand the target setting and participants to include various countries, regions, and different age groups and subsequently compare the results with those of this study. Second, future studies will need to provide more detailed results and wider implications by using qualitative or mixed-method approaches focusing on interviews with students and teachers to provide a complementary reference when interpreting the IPA results. Third, this study derives elements that need improvement and investment in adolescent health education through analysis of their health perception. Therefore, future research is needed to recommend plans to revitalize youth health education, centering on the elements that need further improvement and investment. Fourth, the IPA method produced meaningful results regarding the health perception of Korean adolescents. However, it focused on Quadrants I and III, which represents a limitation of traditional IPA. In future studies, a revised IPA method could enable more diverse analyses through comparison with the results of traditional IPA studies.

## 5. Conclusions

The purpose of this study was to determine the perceived importance and performance of health aspects among South Korean adolescents during the COVID-19 pandemic via an online survey that was conducted in October 2020. We conclude that during the pandemic, Korean adolescents recognized sanitary healthcare and disease as important and practiced relevant healthcare behaviors in their daily lives. For example, they were aware that an infection should be actively treated. However, in terms of prevention and management, they were not aware of various ways to maintain and improve their health. Therefore, when national and public educational institutions seek to maintain and promote adolescent health during the COVID-19 pandemic, they will need to introduce preventive and administrative-level programs to ensure regular participation in physical activity, proper eating habits, and adequate sleep, along with access to other therapeutic services. Second, adolescents in Korea are not very aware of the importance of physical activity and are not practicing this aspect of healthcare in their daily lives. This is more noticeable due to the pandemic, when awareness and practice of physical activities lack compared to developed countries, due to the influence of Korea’s entrance examination-oriented education system. Currently, with the possibility of the pandemic continuing, physical education scholars and researchers are developing programs to perform physical activities at home. Therefore, the Ministry of Education, schools, and related institutions should make efforts to promote physical activity in conjunction with online and offline physical education classes, school sports clubs, and after-school sports activities to actively utilize such programs.

In this study, through the analysis of adolescent health perception, the aspects that need improvement and investment in adolescent health education were derived. These results can be used to prioritize adolescent health education policies in the country. In addition, it is expected that this study will serve as the theoretical basis for the future development of national and school curriculum, as well as planning and carrying out health education in youth-related institutions such as the Ministry of Education and schools.

## Figures and Tables

**Table 1:** Demographic characteristics of the participants.

Characteristics		*n*	%
Sex	Male	153	45.9
	Female	180	54.1
Age	14	108	32.4
	15	98	29.4
	16	127	38.2
Academic achievement grade	Upper rank	98	29.4
	Middle rank	130	39.0
	Lower rank	105	31.5
Physical activity promotion system	1st grade	74	22.2
	2nd grade	89	26.7
	3rd grade	131	39.3
	4th grade	27	8.1
	5th grade	12	3.6
Total		333	

**Table 2:** Reliability analysis.

Variables		Cronbach’s α
Mental health	Importance	0.849
	Performance	0.902
Disease	Importance	0.743
	Performance	0.721
Physical activity	Importance	0.843
	Performance	0.838
Sleep	Importance	0.778
	Performance	0.774
Diet	Importance	0.808
	Performance	0.713
Sanitary health	Importance	0.921
	Performance	0.819

**Table 3:** Descriptive statistics.

Variables		Mean	Rank	Standard Error	Skewness	Kurtosis
Importance	Mental health	4.63	3	0.53	−1.39	1.13
	Disease	4.67	2	0.51	−1.87	3.59
	Physical activity	4.30	6	0.68	−0.83	0.19
	Sleep	4.54	4	0.58	−1.47	3.06
	Diet	4.42	5	0.67	−1.05	0.42
	Sanitary health	4.76	1	0.43	−2.60	7.92
Performance	Mental health	4.00	3	0.82	−0.43	−0.41
	Disease	4.40	2	0.65	−1.02	0.45
	Physical activity	3.62	6	0.95	−0.34	−0.56
	Sleep	3.77	5	0.83	−0.36	−0.26
	Diet	3.89	4	0.82	−0.51	0.01
	Sanitary health	4.51	1	0.47	−1.33	2.04

Note: A five-point Likert scale was used. Rank refers to the ranking of importance and performance for each factor. Skewness represents the direction and degree of inclination of the distribution. Kurtosis refers to the sharpness of the distribution.

**Table 4:** Correlation analysis of health perception importance.

	A	B	C	D	E	F
A	1.000					
B	0.501 ***	1.000				
C	0.533 ***	0.551 ***	1.000			
D	0.584 ***	0.518 ***	0.632 ***	1.000		
E	0.599 ***	0.528 ***	0.659 ***	0.711 ***	1.000	
F	0.472 ***	0.657 ***	0.495 ***	0.486 ***	0.574 ***	1.000

Note: A = mental health, B = disease, C = physical activity, D = sleep, E = diet, F = sanitary health. *** *p* < 0.001.

**Table 5:** Correlation analysis of health perception performance.

	A	B	C	D	E	F
A	1.000					
B	0.499 ***	1.000				
C	0.520 ***	0.343 ***	1.000			
D	0.568 ***	0.517 ***	0.497 ***	1.000		
E	0.583 ***	0.552 ***	0.541 ***	0.648 ***	1.000	
F	0.420 ***	0.575 ***	0.357 ***	0.425 ***	0.492 ***	1.000

Note: A = mental health, B = disease, C = physical activity, D = sleep, E = diet, F = sanitary health. *** *p* < 0.001.

**Table 6:** Analysis of the difference between importance and performance by categories.

Sub-Dimensions of Health Perception	Importance		Performance		Mean Difference	Rank	*t*
	Mean	Standard Deviation	Mean	Standard Deviation			
Mental health #1 (happiness)	4.68	0.60	4.11	0.89	0.57	3	12.021 ***
Mental health #2 (interest in life)	4.60	0.64	3.98	0.93	0.62		13.133 ***
Mental health #3 (life satisfaction)	4.66	0.59	3.97	0.92	0.69		14.683 ***
Mental health #6 (direction of life)	4.57	0.70	3.95	1.00	0.62		12.280 ***
Overall	4.63	0.53	4.00	0.82	0.63		15.665 ***
Disease #1 (disease measures)	4.65	0.68	4.30	0.84	0.35	5	7.808 ***
Disease #2 (doctor prescription	4.73	0.52	4.50	0.71	0.23		7.240 ***
Disease #3 (vaccination)	4.63	0.69	4.41	0.87	0.22		5.321 ***
Overall	4.67	0.51	4.40	0.65	0.27		9.362 ***
Physical activity #1 (cardiovascular exercise	4.23	0.85	3.56	1.23	0.67	2	11.799 ***
Physical activity #2 (daily life exercise	4.30	0.84	3.70	1.18	0.68		10.915 ***
Physical activity #3 (body movement	4.38	0.79	3.79	1.07	0.59		11.515 ***
Physical activity #4 (walking	4.28	0.82	3.44	1.17	0.84		13.677 ***
Overall	4.30	0.68	3.62	0.95	0.68		15.482 ***
Sleep #1 (constant bedtime	4.41	0.82	3.44	1.17	0.97	1	15.790 ***
Sleep #2 (quality of sleep	4.56	0.71	3.32	1.22	1.24		17.733 ***
Sleep #3 (enough sleep)	4.60	0.67	3.96	1.12	0.64		11.515 ***
Sleep #4 (sleeping environment)	4.58	0.76	4.34	0.92	0.24		13.677 ***
Overall	4.54	0.58	3.77	0.83	0.77		19.381 ***
Diet #1 (meals at regular times)	4.45	0.80	3.77	1.14	0.68	4	12.026 ***
Diet #2 (chew enough	4.36	0.83	3.83	1.02	0.53		9.914 ***
Diet #3 (moderate amount of meal	4.46	0.72	4.08	0.91	0.38		7.979 ***
Overall	4.42	0.67	3.89	0.82	0.53		13.665 ***
Sanitary health #1 (wearing a mask	4.87	0.41	4.82	0.55	0.05	6	2.056 *
Sanitary health #2 (no visit to crowded public facilities	4.74	0.58	4.70	0.65	0.04		1.402
Sanitary health #3 (social distancing)	4.79	0.52	4.52	0.72	0.27		7.773 ***
Sanitary health #4 (using hand sanitizer)	4.65	0.62	4.09	1.00	0.56		12.462 ***
Sanitary health #5 (cough etiquette)	4.75	0.56	4.49	0.74	0.26		8.198 ***
Sanitary health #6 (hand hygiene)	4.61	0.69	3.95	1.00	0.66		13.837 ***
Sanitary health #7 (washing hands	4.74	0.61	4.50	0.74	0.24		6.985 ***
Sanitary health #8 (washing hands after returning home	4.81	0.51	4.66	0.60	0.15		5.410 ***
Sanitary health #9 (wearing a mask in a medical institution)	4.85	0.43	4.83	0.43	0.19		0.904
Overall	4.76	0.43	4.51	0.47	0.25		15.041 ***

Note: paired-samples *t*-tests between importance and performance of dimensions. The five-point Likert scale was used. * *p* < 0.05, *** *p* < 0.001.

**Table 7:** Distribution of dimensions of health perception.

Quadrant	Characteristics	Health Perception Dimensions
Quadrant I	Importance↑, performance↑	Sanitary health, disease
Quadrant II	Importance↑, performance↓	Mental health
Quadrant III	Importance↓, performance↓	Physical activity, sleep, diet
Quadrant IV	Importance↓, performance↑	None

## Data Availability

The data presented in this study are available on request to the authors.

## References

[1] World Health Organization (2020). Coronavirus Disease (COVID-19) Advice for the Public. Corona Virus Disease..

[2] Ciotti M., Ciccozzi M., Terrinoni A., Jiang W.C., Wang C.B., Bernardini S. (2020). The COVID-19 pandemic. Crit. Rev. Clin. Lab. Sci..

[3] Holmes E.A., O’Connor R.C., Perry V.H., Tracey I., Wessely S., Arseneault L., Ballard C., Christensen H., Silver R.C., Everall I. (2020). Multidisciplinary research priorities for the COVID-19 pandemic: A call for action for mental health science. Lancet Psychiatry.

[4] Condello G., Capranica L., Stager J., Forte R., Falbo S., Di Baldassarre A., Segura-Garcia C., Pesce C. (2016). Physical activity and health perception in aging: Do body mass and satisfaction matter? A three-path mediated link. PLoS ONE.

[5] Curi V.S., Vilaça J., Haas A.N., Fernandes H.M. (2018). Effects of 16-weeks of Pilates on health perception and sleep quality among elderly women. Arch. Gerontol. Geriatr..

[6] World Health Organization (1992). Basic Documents.

[7] Ware J.E. (1979). Health Perception Questionnaire Instruments for Measuring Nursing Practice and other Care Variables.

[8] Izadi A., Jahani Y., Rafiei S., Masoud A., Vali L. (2017). Evaluating health service quality: Using importance performance analysis. Int. J. Health Care Qual. Assur..

[9] Lopes S.D.F., Maia S.C.F. (2012). Applying importance-performance analysis to the management of health care services. China-USA Bus. Rev..

[10] Miranda F.J., Chamorro A., Murillo L.R., Vega J. (2010). An importance-performance analysis of primary health care services: Managers vs. patients perceptions. J. Serv. Sci. Manag..

[11] Rau H.H., Wu Y.S., Chu C.M., Wang F.C., Hsu M.H., Chang C.W., Chen K.H., Lee Y.L., Kao S., Chiu Y.L. (2017). Importance-performance analysis of personal health records in Taiwan: A web-based survey. J. Med Internet Res..

[12] Stevenson J.P. (2002). Applied Multivariate Statistics for the Social Sciences.

[13] Barakat R., Pelaez M., Montejo R., Luaces M., Zakynthinaki M. (2011). Exercise during pregnancy improves maternal health perception: A randomized controlled trial. Am. J. Obstet. Gynecol..

[14] Jones A.L. (2018). The influence of shape and colour cue classes on facial health perception. Evol. Hum. Behav..

[15] Lee S.M., Jeong H.C., So W.Y., Youn H.S. (2020). Mediating Effect of Sports Participation on the Relationship between Health Perceptions and Health Promoting Behavior in Adolescents. Int. J. Environ. Res. Public Health.

[16] Weaver B., Maxwell H. (2014). Exploratory factor analysis and reliability analysis with missing data: A simple method for SPSS users. Quant. Methods Psychol..

[17] Martilla J.A., James J.C. (1977). Importance-performance analysis. J. Mark..

[18] Kline R.B. (2015). Principles and Practice of Structural Equation Modeling.

[19] West S.G., Finch J.F., Curran P.J. (1995). Structural Equation Models with Nonnormal Variables: Problems and Remedies.

[20] Kim B.W. (2015). Economic Value Measurement and IPA Analysis Method.

[21] Ainin S., Hisham N.H. (2008). Applying Importance-Performance Analysis to Information Systems: An Exploratory Case Study. J. Inf. Inf. Technol. Organ..

[22] Jefsen O.H., Rohde C., Nørremark B., Østergaard S.D. (2020). Editorial Perspective: COVID-19 pandemic-related psychopathology in children and adolescents with mental illness. J. Child Psychol. Psychiatry.

[23] Brain Editorial Department (2020). The Age of Uncontact, The Rise of Well-Being and Managing Living Havits: The Change in the Perception of Health brought by COVID-19. Brain.

[24] Mostert S., Kesselring J. (2002). Effects of a short-term exercise training program on aerobic fitness, fatigue, health perception and activity level of subjects with multiple sclerosis. Mult. Scler. J..

[25] Bae Y.J., Hyeon I.S. (2016). Sports Activity and Subjective Health Recognition of the Elderly in Community Senior Centers. Korean J. Sport.

[26] Henchoz K., Cavalli S., Girardin M. (2008). Health perception and health status in advanced old age: A paradox of association. J. Aging Stud..

